# Laser-Assisted aPDT Protocols in Randomized Controlled Clinical Trials in Dentistry: A Systematic Review

**DOI:** 10.3390/dj8030107

**Published:** 2020-09-22

**Authors:** Valina Mylona, Eugenia Anagnostaki, Steven Parker, Mark Cronshaw, Edward Lynch, Martin Grootveld

**Affiliations:** 1 Leicester School of Pharmacy, De Montfort University, Leicester LE1 9BH, UK; eanagnostaki@densindente.de (E.A.); thewholetooth@easynet.co.uk (S.P.); drmarkcronshaw@outlook.com (M.C.); edward.lynch@hotmail.com (E.L.); mgrootveld@dmu.ac.uk (M.G.); 2 School of Dentistry, College of Medical and Dental Sciences, Birmingham University, Birmingham B15 2TT, UK; 3 School of Dental Medicine, University of Nevada Las Vegas, Las Vegas, NV 89154, USA

**Keywords:** aPDT, dentistry, laser, parameters, PDT, photodynamic therapy

## Abstract

Background: Antimicrobial photodynamic therapy (aPDT) has been proposed as an effective alternative method for the adjunctive treatment of all classes of oral infections. The multifactorial nature of its mechanism of action correlates with various influencing factors, involving parameters concerning both the photosensitizer and the light delivery system. This study aims to critically evaluate the recorded parameters of aPDT applications that use lasers as the light source in randomized clinical trials in dentistry. Methods: PubMed and Cochrane search engines were used to identify human clinical trials of aPDT therapy in dentistry. After applying specific keywords, additional filters, inclusion and exclusion criteria, the initial number of 7744 articles was reduced to 38. Results: Almost one-half of the articles presented incomplete parameters, whilst the others had different protocols, even with the same photosensitizer and for the same field of application. Conclusions: No safe recommendation for aPDT protocols can be extrapolated for clinical use. Further research investigations should be performed with clear protocols, so that standardization for their potential dental applications can be achieved.

## 1. Introduction

The discovery of penicillin by Alexander Fleming in 1928 was one of the scientific highlights of the last century. In the 1940s, antibiotics had been introduced to the market and in the 1980s, pharmaceutical companies were declaring the “end” of infectious diseases. Unfortunately, microorganisms remained, and the extensive and inappropriate use of antibiotics gradually led to the development of pervasive antimicrobial resistance. Since the efficacies of antibiotics decreases and the end of the “antibiotic era” gets closer, efforts to discover new ways to eradicate microorganisms and eliminate multidrug resistance phenomena are evolving. Photodynamic therapy (PDT) therefore serves as a promising approach [1].

Photodynamic therapy is a non-thermal photochemical reaction that involves the excitation of a non-toxic dye (photosensitizer-PS) by light at an appropriate wavelength, to produce a long-lived triplet state that can interact with molecular oxygen to produce reactive oxygen species (ROS), including singlet oxygen (^1^O_2_), which can damage biomolecules, such as polyunsaturated fatty acids [2]. Each of the above-mentioned components (photosensitizer, light and oxygen) are harmless by themselves, but in combination lead to lethal cytotoxic ROS that can selectively destroy cells [3]. This therapy affects the target tissue, which is exposed both to a light source and photosensitizer simultaneously. It shows a dual selectivity, which is based on the different concentrations of the photosensitizer used between normal and target tissue, and also on the spatial confinement of the light only in the target [4].

Photosensitizers are usually organic aromatic molecules with delocalised π electrons, where a central chromophore is covalently bonded to auxiliary substituent branches, which contribute to further electron delocalisation. In this manner, the absorption spectrum of the photosensitizer moiety is modified [5]. They should absorb light at the red or near-infrared wavelengths (600–800 nm). Shorter wavelengths (i.e., those <600 nm) have less penetration and longer wavelengths (i.e., >800 nm) do not have sufficient inherent photonic energy to interact with and induce photodynamic reactions [6].

The source of light must coincide with the absorption maximum of each photosensitizer used. Devices that can be employed include broad-spectrum lamps, light-emitting diodes (LED) or lasers. Amongst these, lasers have specific properties, which render them superior to the other sources. Monochromaticity is a unique and inherent characteristic that provides the laser with the possibility to interact with the photosensitizer by accurately matching its peak absorption. This results in less excess energy and tissue heating, which is sub-optimal in delivering the PDT reaction, when compared to the effects of broad bandwidth devices [7].

The main advantages of PDT are the wide spectrum of antimicrobial action; treatment outcomes are independent of the antibiotic resistance pattern, minimal damage to host tissue, the absence of photo-resistant strains of microorganisms after multiple treatments, a lack of mutagenicity, and minimally invasive and low-cost therapies [8].

Photodynamic therapy has been widely applied for cancer therapy in general medicine. Notwithstanding this, today the interest for antimicrobial PDT has increased in view of the consequences experienced with antibiotic overuse [8]. Several acronyms exist to describe this therapy and in order to avoid any confusion with photodynamic therapy applied for tumour treatments, antimicrobial photodynamic therapy (aPDT) is the most suitable term for antimicrobial purposes [9], as applied in dentistry.

The use of aPDT in dentistry can be readily justified, since the oral cavity is heavily populated with microorganisms, organised within biofilm structures that may show extremely high resistance to conventional antimicrobial agents [1]. Additionally, the uncontrolled systemic use of antibiotics has led to highly resistant microorganisms [10]. Thus, the investigation of an alternative potential treatment for local infections, such as photodynamic therapy, is mandated [11].

The mechanism of action of aPDT can be explained in the following manner: the ground electronic state of the photosensitizer is a singlet state, since it has two electrons paired with opposite spins within its external molecular orbital (highest occupied molecular orbital—HOMO). When the photosensitizer absorbs the appropriate quantum energy from a light source, one of these two electrons is excited to a higher-energy orbital (lowest unoccupied molecular orbital—LUMO). This is termed the first excited singlet-state [12]. To absorb a photon, the energy of the incident photon should be equal or higher than the HOMO–LUMO energy gap and the excess of energy is released through vibrational relaxation; on return to its ground state, the photosensitizer emits the absorbed energy as fluorescence, or produces heat by internal conversion, which is a non-radiative and rapid (less than a nanosecond) process in which electron spins remain the same [8]. Alternatively, the excited singlet-state photosensitizer can undergo a process known as “intersystem crossing” to form a more stable, first excited triplet state. Again, this process is non-radiative and involves a change in spin for the excited electron, so the photosensitizer now has two unpaired but parallel electrons [13]. This endures for <10 ns [8], and the excited triplet state has a lifetime of microseconds [2], so there is sufficient time to induce photochemical reactions. The triplet state also has a lower energy than the excited singlet state [1].

If there is no molecular oxygen (O_2_) available, the triplet state photosensitizer can eventually return to the ground state through internal or external fluorescence or phosphorescence [13]. However, in the presence of O_2_, the triplet excited state photosensitizer can participate in chemical reactions and provide photodynamic therapy. Indeed, there are two types of these reactions—Type I and Type II [2]. In Type I, hydrogen and electron transfers take place between the triplet excited state of the photosensitizer and other molecules, predominantly O_2_. With these chemical reactions, reactive oxygen species (ROS) are produced, that are very active and harmful towards many target cells [13]. These ROS predominantly consist of superoxide anion (O_2_^●−^), hydrogen peroxide (H_2_O_2_), hydroxyl radical (^●^OH), and singlet oxygen (^1^O_2_) [2]. However, the converse, Type II reaction is much simpler, and involves energy transfer between the triplet state photosensitizer and O_2_. This results in the formation of ground state photosensitizer and ^1^O_2_ [2].

Singlet oxygen and ^●^OH radical can readily pass through cell membranes and are the most highly reactive ROS species. In view of this, only molecules that are closely located to their site of generation can be affected by photodynamic therapy [6]. Additionally, the lifetime of singlet oxygen (^1^O_2_) is very limited, depending on the surrounding solvent present [14], thus its action radius is approximately 10–55 nm [12]. Hence, the most important factor that influences the outcome of photodynamic therapy is the subcellular localisation of the photosensitizer which drives the process.

In general, the efficiency of the treatment can be affected by the following factors [6]:

- As noted above, the sub-cellular localisation of the photosensitizer. Within the target cell, the photosensitizer may affect lysosomes, mitochondria, the plasma membrane, Golgi apparatus and the endoplasmic reticulum. Most of the photosensitizers localise within mitochondria, where apoptosis is provoked via mitochondrial damage; lysosomes accumulate photosensitizers with more aggregation. The photosensitizer Foscan (a chlorin named *m*-tetrahydroxyphenylchlorin) may target the Golgi apparatus and the endoplasmic reticulum [6]. However, the plasma membrane is rarely noted as a site of photosensitizer accumulation [10].
- The chemical characteristics of the photosensitizer. The different physiology of Gram-positive and Gram-negative bacteria can affect the degree of binding of different photosensitizers. Indeed, Gram-positive bacteria can efficiently bind to cationic, neutral and anionic photosensitizers, while only cationic ones can bind to Gram-negative bacteria [15].
- The concentration of the photosensitizer applied. High concentrations of photosensitizer can be naturally cytotoxic in a non-illuminated state, and obstruct light transmission into tissue target sites [16].
- The blood serum content. The presence of serum in the medium can decrease the effectiveness of the therapy, in view of probable chemical and physicochemical interactions between such agents and selected serum biomolecules [17].
- The incubation time, also known as equilibration time, of the photosensitizer at target sites. This should ideally commence shortly prior to illumination (of a ca. a few minutes’ duration), since this favours localisation into the microorganisms, and does not allow penetration into host cells (this process requires many hours to occur) [18].
- The phenotype of the target cell. It is known that different tissue types have differential light optical properties of light (i.e., absorption and scattering) [6].

An understanding of the mode of action of antimicrobial photodynamic therapy, and a knowledge of the structure of the target host tissue is essential. This should facilitate determination of the correct choice of photosensitizer (type, concentration, incubation time, etc.), and the correct light source (kind, power, illumination time, energy, spot size, distance from the target, technique applied, etc.) in order to produce a standardized protocol.

In the scientific literature, a variety of reports exist regarding the use of aPDT in dentistry. This technique has been tested in the treatment of periodontitis, peri-implantitis, endodontic conditions, dental caries and candida disinfection, wound healing and oral lichen planus (OLP). For the latter, photodynamic therapy has been suggested as an alternative treatment based on the inflammatory pathogenesis of OLP and the immunomodulatory effect of aPDT [19].

However, until now there is no consensus regarding the protocol to be applied. The aim of this study is to critically evaluate, by a systematic review of randomized clinical trials, the recorded parameters of laser aPDT applications in clinical dentistry and oral health.

## 2. Materials and Methods

### 2.1. Search Strategy

An electronic search was conducted relating to aPDT applications in all fields of dentistry from 10 March until 20 March. Databases used were PubMed and Cochrane, with the following MeSH terms, keywords and their combinations: (1) (PDT OR aPDT OR photodynamic) AND laser; and (2) photodynamic AND (periodontitis OR peri-implantitis OR endodontic OR caries OR candida OR oral lichen OR halitosis).

After applying the additional filters (published within the last 10 years, only randomized clinical trials in humans, and only English language reports), the preliminary number of 7744 articles was reduced to 390.

Titles and abstracts of the above articles were independently screened by two reviewers via application of the following criteria. In case of any disagreements arising, these were satisfactorily resolved by discussions.

Inclusion criteria:

- laser used as light source;
- negative control group;
- at least 10 samples/patients per group;
- only randomized controlled clinical trials;
- correct combinations of photosensitizer (PS) and the laser source employed;
- a minimum of a 6 month follow-up for periodontitis/peri-implantitis articles.

Exclusion criteria:

- duplicates or studies with the same ethical approval number;
- tumours, general medical applications, aPDT form not used as a therapy;
- LED or lamps used as light sources;
- no negative control group;
- low sample/patients sizes (less than 10 per group);
- no randomized controlled clinical trials or pilot studies;
- erroneous combinations of photosensitizer and laser employed;
- for periodontitis/peri-implantitis articles:
  ➢ <6 month follow-up
  ➢ aPDT used as a monotherapy (without scaling and root planning—SRP)

After screening and implementation of the eligibility criteria, a total of 38 articles were retained. These concerned a range of different aspects of application fields in dentistry. Specifically, the number of articles per field was found to be:

- periodontitis: 17
- peri-implantitis: 4
- endodontics: 5
- caries disinfection: 5
- candida disinfection: 2
- halitosis: 1
- oral lichen planus (OLP): 3
- healing of pericoronitis: 1

**Figure 1:**
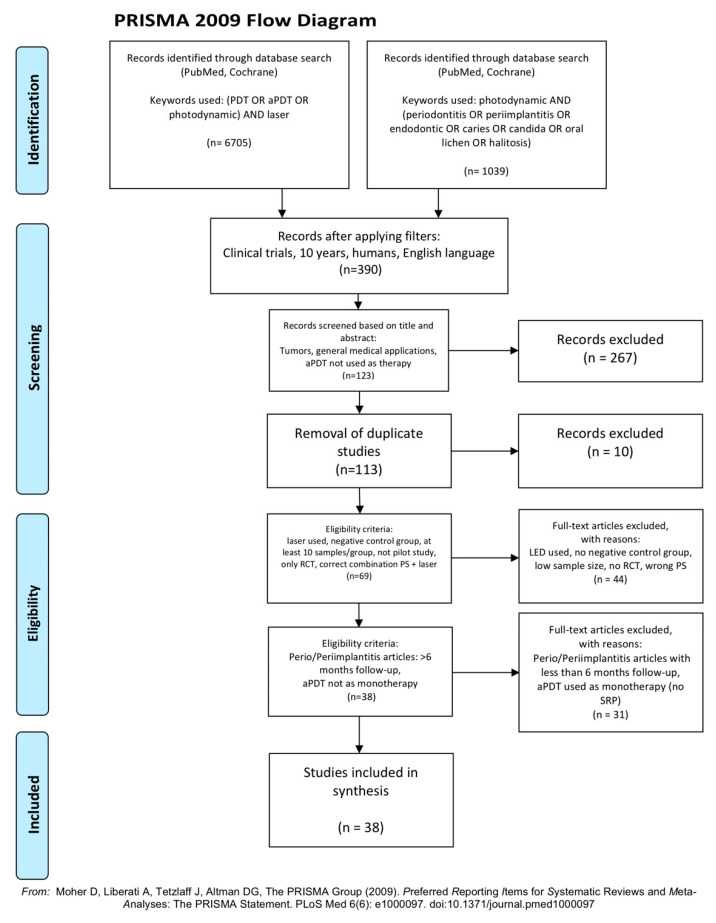
PRISMA flow-chart of selected criteria for the included article reports [20].

In accordance with the PRISMA statement [20], details of the selection criteria are presented in Figure 1.

### 2.2. Data Extraction

Having reached a consensus regarding the selection of included articles, the two reviewers involved subsequently extracted data regarding:

- Citation (first author and publication year);
- Type of study/number of samples/pocket depth (only for periodontitis and peri-implantitis articles);
- Test/control groups;
- Laser and photosensitizer used (PS concentration);
- aPDT protocol/number of sessions involved;
- Follow-up;
- Outcome.

### 2.3. Quality Assessment

Subsequent to data extraction, articles were further evaluated by assessing their risk of bias assessment. The Cochrane Risk of Bias tool [21] was modified according to the requirements of this systematic review.

The risk of bias was determined according to the number of “yes” or “no” responses to the parameters provided below, which were allocated to each study:

- Randomization?
- Sample size calculation and required sample numbers included?
- Baseline situation similar to that of the test group?
- Blinding?
- Parameters of laser use described appropriately, and associated calculations correct?
- Power meter used?
- Numerical results available (statistics)?
- No missing outcome data?
- All samples/patients completed the follow-up evaluation?
- Correct interpretation of data acquired?

The classification was performed according to the total number of “yes” answers to the above questions. For the current study, the degree of bias was computed according to the score limits provided below:

1. High risk: 0–4
2. Moderate risk: 5–7
3. Low risk: 8–10

## 3. Results

### 3.1. Primary Outcome

The primary goal of this systematic review was to evaluate the studies explored with sufficient and reproducible parameter descriptions, and also analyse their aPDT protocols.

The parameters missing from the studies with incomplete protocols are also briefly noted.

### 3.2. Data Presentation

The extrapolated data evaluated for each dental research field are presented in Table 1, Table 2, Table 3, Table 4, Table 5, Table 6 and Table 7. Key: TBO—Toluidine Blue, MB—Methylene Blue, ICG—Indocyanine Green.

### 3.3. Quality Assessment Presentation

The risk of bias of the included studies is presented in Table 8.

In total, 21/38 of the articles (55.3%) showed a low risk of bias, with two articles [6,31] scoring 10/10, eleven [24,26,28,29,38,43,44,49,51,52,55] scoring 9/10, and eight [23,25,34,35,37,46,48,50] scoring 8/10.

Respectively, 17/38 of the articles (44.7%) showed a moderate risk of bias, with ten articles [22,27,32,33,39,42,47,53,58,59] scoring 7/10, and seven [30,40,41,45,54,56,57] scoring 6/10.

Overall, the mean ± standard error (SEM) Cochrane risk of bias score parameter was 7.76 ± 0.20 out of a perfect, optimal value of 10.

Apart from the correct description of the aPDT protocol, the most common negative answers concerned (a) use of a power meter, and (b) the sample size power calculation and required sampling numbers included.

### 3.4. Analysis of Data

Regarding the primary outcome, 22/38 articles (57.9%) presented an appropriate and sufficient description of the aPDT protocol used.

Specifically, for each dental research field, studies were allocated as:

- 8/17 in periodontitis [22,24,26,28,29,31,36,38];
- 2/4 in peri-implantitis [39,41];
- 4/5 in endodontics [43,44,46,47];
- 5/5 in caries disinfection [48,49,50,51,52];
- 0/2 in candida disinfection;
- 1/1 in halitosis [55];
- 2/3 in OLP [56,58];
- 0/1 in healing pericoronitis.

From these studies, 16/22 showed a low risk of bias, whilst 6/22 showed a moderate risk level.

The analysis of the aPDT protocols have been performed for each photosensitizer used, as listed in Table 9, Table 10, Table 11 and Table 12:

For investigations with incomplete parameter descriptions, 16/38 present the following deficiencies, as noted from Table 1, Table 2, Table 3, Table 4, Table 5, Table 6 and Table 7:

- incubation time: 2/16 (12.5%);
- power: 4/16 (25%);
- tip or spot size: 13/16 (81.2%);
- fluence value incorrectly calculated (i.e., either the tip or energy applied is erroneous): 2/16 (12.5%).

## 4. Discussion

Data analysis of the publications reviewed revealed a considerable variety in the report of parameters concerning the use of aPDT treatments in different dental fields. This is in accordance with Parker et al. [60], and points out the necessity to adopt clear information on the materials and methods. We then considered studies with an appropriate description of aPDT protocols, specifically those which indicated, or allowed us to calculate, the following parameters: power, irradiation time, total energy delivered, tip diameter or spot size at target tissue, any movement and speed of movement, the photosensitizer used, its applied concentration, its incubation time, and finally protocols available for washing it away or not prior to illumination. The ideal reporting of an aPDT protocol is indicated in Table 13.

An important aspect to be considered is the use of a power meter prior to the illumination process. Indeed, the laser should be calibrated in order for investigators to obtain precise parameters to record, so that a standardised protocol can be provided [61]. In this review, only 6/38 [29,31,36,51,52,55] articles used a power meter (Table 8).

With regard to the treatment outcomes observed in the surveyed investigations, only 2/38 studies showed negative results when expressed relative to those of their corresponding control groups. The remainder of the investigations showed either positive (22/38) or indifferent (14/38) result outcomes when compared to results acquired for their corresponding control groups. This heterogeneity can be mainly attributed to the different protocols applied (i.e., either laser or photosensitizer parameters, as described above). Moreover, other factors that should be considered are the complex pocket or root canal architecture, unknown total volume irradiation of the photosensitizer, and the variable numbers of treatment sessions employed by investigators.

### 4.1. aPDT Components

As noted in the introduction, antimicrobial photodynamic therapy is based on the combination of three components: the photosensitizer nature, light and O_2_ [2]. Basic information available on each of these considerations is further analysed below.

#### 4.1.1. Photosensitizers

The vast majority of articles used methylene blue (MB) as the photosensitizer, which has an absorption band located at 660 nm. It is a cationic and hydrophilic compound, i.e., an amphipathic molecule (one that combines both polar and non-polar moieties), which has a low molecular mass [3]. In view of its charge, it can bind to the lipopolysaccharides of the outer membrane of Gram-negative bacteria, and also to the teichuronic acid residues of the outer membrane of Gram-positive bacteria [7].

Another popular photosensitizer is toluidine blue (TBO), with an absorption band centred at 635 nm [7]. It is a blue colouring agent also with amphipathic characteristics, but with a positive charge and a hydrophilic portion [62]. In view of its charge, it can bind both to Gram-positive and Gram-negative bacteria [7], as documented above.

The other photosensitizer used in studies included in this review is indocyanine green (ICG). It is a green colouring agent, with anionic charge, and also has amphiphilic characteristics; indeed, its polycyclic components are lipophilic [9]. It has an absorption band with a maximum at 810 nm (although this precise value is critically dependent on the dissolution medium employed), its concentration and extent of binding to blood plasma proteins [7]. Notably, its mechanism of action is predominately based on photothermal (80%) rather than photochemical (20%) processes [63].

The final photosensitizer included is the chlorin(e6) conjugate of polyethyleneimine (PEI-ce6). It is a polycationic macromolecule, and its treatment efficacy is dependent on the molecular size (smaller values lead to greater diffusion into cells), and the cationic charge (the higher the charge, the more effective it is). As expected, its absorption spectrum in the visible region of the electromagnetic spectrum is the same as that of the free chlorin(e6) conjugating agent with absorption maxima located at 400 and 670 nm [64,65].

Unfortunately, studies with curcumin, 5-aminolevulinic acid, rose Bengal and erythrosine used as photosensitizers have not been included, since they failed to meet the inclusion criteria of this review. To date, there are no published human clinical trials using 5-aminolevulinic acid, rose Bengal and erythrosine as photosensitizers in the dental fields. Notwithstanding, for curcumin, there are recent human clinical trials that reported using LEDs as the light source, and with promising results obtained [66,67,68,69,70,71].

#### 4.1.2. Light Diffusion

Light distribution depends on the shape of the beam [72]; thus, diffusor tips, as used in the included studies [22,33,40,41,42,46], are preferable since they lead to a three-dimensional illumination [73]. As Garcez et al. pointed out, the use of a conventional tip inside the root canal will lead to ROS generation in the middle of the canal, and not inside the dentin walls, where most of the microorganisms are located [74].

Furthermore, the optical properties of the target tissue play a crucial role regarding the diffusion of light. As noted in [72], these can be identified as (a) different refraction and scattering indexes when light passes through differing media, as previously noted for trans-gingival use [75]; (b) competitive light absorbers; and (c) unevenly distributed absorbers, since the photosensitizer can lead to local “cold spots” as far as the applied irradiance is concerned [72].

Regarding the use of trans-gingival as an aPDT, as applied in studies [36,38] evaluated here, such a therapy may be considered a novel approach, and this approach appears to be able to bypass the limitation of light in accessing complex target areas, such as root furcations or deep periodontal pockets [76,77]. It is known that the penetration depth of the 660 nm wavelength is 3–3.5 mm, while that for the range of 800–900 nm is 6–6.5 mm [76]. However, it is essential to consider that light attenuation occurs within gingival tissue. Specifically, for red light at a depth of 3 mm inside the gingival tissue, there is a 50% loss of intensity [75].

With regard to the competitive host absorbers of light, such as haemoglobin and a wide range of other proteins, it is mandatory to consider that their presence can decrease the effectiveness of the therapy applied [17,78]. Therefore, the outcome should be carefully evaluated when the aPDT technique is applied immediately after the SRP or pocket debridement, as was indeed the case in the majority of the studies included here for periodontitis and peri-implantitis treatment (13/21). Respectively, in endodontic therapy, the root canals should be dried prior to application of the photosensitizer. The photosensitizers used within a confined space, i.e., a root canal or a periodontal pocket, are investigated at a precise, pre-calculated concentration. If, for any reason, this space is not “dry”, the photosensitizer may not achieve the concentration required for its optimal activity.

Higher concentrations of photosensitizer applied can lead to limitations in its ability to absorb light, either by the “photobleaching” phenomenon [79], or alternatively the “optical shielding” effect [6]. The former occurs when ROS generated chemically react with the photosensitizer, as noted above, and hence circumvents any further photosensitization process [79]. The latter refers to the blocking of light in view of high superficial absorption, and prevention of the light from reaching deeper tissue layers [74].

The above mentioned three photosensitizers (MB, TBO and ICG) can be considered to be ROS-scavenging antioxidant molecules [79].

#### 4.1.3. Oxygen

Sufficient oxygenation of the target tissue is crucial for inducing and propagating the direct oxidative damage of microorganisms [80]; in deep and less oxygenated areas, such as in root canals, there is an O_2_ deficiency. To surmount this hurdle, firstly ICG, with its photothermal action, can be used to enhance the elimination of microorganisms, although thermal damage to surrounding tissues should be taken into consideration [81]. Secondly, pre-treatment of root canals with H_2_O_2_ has been suggested. This will enhance O_2_ availability in this environment and allow an improved penetration of the photosensitizer inside microbial biofilms, a process leading to a higher level of antimicrobial effectiveness [74].

### 4.2. Healing

The healing of tissues is known to be improved following photodynamic therapy, rendering this treatment regimen a valuable choice for wounds or other infections. An additional consideration is that in many local infections, the photosensitizer is topically administered to the infected area, and the delivered light diffuses and scatters well beyond the actual area of interest. This light can exert a substantial secondary therapeutic beneficial effect in stimulating healing and repair within the surrounding tissues by a process known as photobiomodulation (PBM) [18]. Even if the whole of the photosensitizer dye solution cannot be activated, the benefits offered by PBM are invaluable [76].

### 4.3. Clinical Aspects

The most investigated and effective photosensitizer is methylene blue; indeed, it was applied in a total of 29 out of the 38 studies included in the present review applied MB as the photosensitizer. Nevertheless, ICG is a very promising agent, since it is activated by an 810 nm laser, which can penetrate deeper into tissues, and therefore, trans-tissue illumination is possible. In addition, in view of its additional photothermal actions (80%), applications inside root canals, where oxygen is limited, are preferential.

However, to date there is no ideal PS available, and hence clinicians should bear in mind the following characteristics before making their choice [13]:

- Selectivity for prokaryotic cells over eukaryotes, so that collateral damage to healthy tissue is minimised;
- Short incubation time, so that binding selectivity is achieved;
- High quantum yields for photochemical reactions and low quantum yields for photobleaching;
- High extinction coefficient, which demonstrates the ability of a molecule to absorb light at a specific wavelength (usually at the maximum absorption band) [8];
- Possess cationic charge and therefore be effective against both Gram-positive and Gram-negative microorganisms;
- Ability to kill multiple kinds of microorganisms at low concentrations and at low light fluences;
- Low side effects, such as photosensitivity and pain;
- Low dark toxicity without applied illumination;

As far as the light dose is concerned, it should be noted that high fluence irradiation will lead to the depletion of molecular oxygen into the tissue, and this will give rise to an impairment of therapy efficacy [13].

From the included studies with appropriate and sufficient description of the aPDT protocol applied (Table 9, Table 10, Table 11 and Table 12), the authors suggest that the power and incubation time of PS should not exceed 200 mW and 5 min, respectively (only two studies [56,58] used a 10 min duration with coupled TBO) and that the irradiation time should not be less than 30 s.

All the above have the prerequisite that the clinician has understood the mechanism of action of photodynamic therapy and its influencing factors outlined in the introduction section, and can therefore select the correct combinations of the photosensitizers and lasers for upcoming dental treatments.

## 5. Conclusions

Photodynamic therapy has been acknowledged to effectively eliminate microorganisms and enhance tissue healing processes. The scope of this systematic review was to critically appraise the recorded aPDT protocols in current clinical trials featuring this form of therapy. Almost half of the articles presented incomplete parameters, whilst the remainder had differential protocols, even with the same photosensitizer and for the same field of application. Consequently, no safe recommendation on aPDT protocols can be extrapolated for clinical use at this point in time.

Unfortunately, light dosimetry is still not widely embraced in clinical aPDT. The main reason for this may be that the effects and benefits of photomedicine are multifactorial, and that the high levels of mathematics, physics and optical technologies are not easily incorporated into clinical practices and their research investigations.

For future directions, more research studies should be performed with clear, validated protocols, so that standardisation in a range of dental applications may be achieved.

## Figures and Tables

**Table 1:** Studies of aPDT in periodontitis.

Citation [ref]	Type of Study/Number of Samples/Pocket Depth	Test/Control Groups	Laser + PS Used (PS Concentration)	aPDT Protocol/Number of Sessions	Follow-Up	Outcome
Grzech-Lesniak et al. (2019) [22]	Parallel-group RCT/40 patients/one pocket with PD ≥ 5 mm, Chronic peridontitis	SRP + PDT (20 patients)/SRP (20 patients)	635 nm + TBO (1 mg/mL)	One minute incubation time, wash with water, 200 mW, CW, 800 μm tip, diffusor tip, 30 s irradiation per pocket. sweeping movement, 117.64 J/mm^2^/3 sessions: 0, 7, 14 days	6 months	No significant difference between groups in PD, PI, CAL, GR. PDT + SRP group sig. difference *p* = 0.007 in BOP and total bacterial count except A.a.
Gandhi et al. (2019) [23]	Split-mouth RCT/26 patients/one pocket with PD ≥ 5 mm in each quadrant, Chronic periodontitis	SRP + PDT (1)/SRP + LLLT (2), SRP alone (two quadrants) (3)	810 nm + ICG (unknown concentration)	Two minutes incubation time, rinsing after with saline, 100 mW, 60 s irradiation inside pocket and upward movement, 60 s irradiation over outer gingiva/1 session: day 0	9 months	Groups 1 and 2 significantly better results than group 3 in P.g. and A.a. pathogen reduction, PI, GI, CAL, PD Groups 1 and 2 no difference
Hill et al. (2019) [24]	Split-mouth RCT/20 patients/one single and one multi-rooted tooth with PD ≥ 4 mm in each quadrant, Chronic periodontitis	SRP + PDT/SRP	808 nm + ICG (0.1 mg/mL)	One minute incubation time, wash with water, 100 mW average, 2 kHz, 300 μm tip, 20 s irradiation, 2829 J/cm^2^ dose per tooth (4sites)/1 session: day 0	6 months	No significant difference between the groups in BOP, PD, GR, CAL and pathogen reduction
Bechara et al. (2018) [25]	Parallel split-mouth RCT/36 patients/one site in each quadrant with PD and CAL ≥ 5 mm and BOP, Aggressive periodontitis	SRP + PDT, SRP + PDT + clarithr. (18 patients)/SRP, SRP + clarithr. (18 patients)	660 nm + MB (10 mg/mL)	One minute incubation time, wash with water, 60 mW, 60 s irradiation per site, 129 J/cm^2^ dose/1 session: day 0	6 months	Significant difference in PD and residual pockets only to antibiotics groups (PDT or not)
Theodoro et al. (2018) [26]	Parallel-group RCT/51 smoking patients/one tooth with PD ≥ 5 mm and one tooth with PD ≥ 7 mm in each quadrant, Chronic periodontitis	SRP + PDT (15 patients)/SRP + antibiotics MTZ + AMX (14 patients), SRP (14 patients)	660 nm + MB (10 mg/mL)	One minute incubation time, 100 mW, spot size 0.03 cm^2^, 48 s irradiation per pocket, 160 J/cm^2^, 4.8 J/3 sessions: day 0, 2, 4	6 months	SRP + PDT significant difference in CAL compared to SRP SRP + PDT and SRP + antibiotics significant reduction in the number of pockets No significant difference between SRP + PDT and SRP + antibiotics groups
Segarra et al. (2017) [27]	Parallel-group RCT/20 healthy patients and 37 with periodontitis/four pockets with PD ≥ 5 mm and BOP, Chronic periodontitis	SRP + PDT (19 patients)/SRP (18 patients), healthy no treatment (20 patients)	670 nm + MB (0.05 mg/mL)	Manufacturer’s instructions, 150 mW, 60 s irradiation each pocket/3 sessions: week 1, 5 and 13	6 months	No significant difference in CAL, PI, PD, GR, BOP, reduction in P.g. and T.f., no pathogen reduction in T.d., P.i., C.rectus aPDT + SRP significant difference in A.a.
Tabenski et al. (2017) [28]	Parallel-group RCT/45 patients/four teeth with PD ≥ 6 mm, Chronic periodontitis	SRP + PDT (15 patients)/SRP + minocycline (15 patients), SRP (15 patients)	670 nm + MB (10 mg/mL)	Manufacturer’s instructions, 3 min incubation time, wash with saline, 75 mW/cm^2^, 6 sites per tooth, 10 s irradiation per site (60 s per tooth)/2 sessions: day 0, 7	12 months	No significant difference between groups in PPD, CAL, BOP A.a, P.g, T.f, T.d
DaCruz et al. (2017) [29]	Parallel-group RCT/28 patients/pockets with PD ≥ 4 mm, Chronic periodontitis	SRP + PDT (14 patients)/SRP (14 patients)	660 nm + MB (0.1 mg/mL)	Five minutes incubation time, washed with water, 200 μm tip, 40 mW, 90 s irradiation per pocket, upward movement, 90 J/cm^2^ dose, powermeter used/1 session: week 6	12 months	No significant difference between groups in PD CAL, BOP, PI. IL-1α and IL-1β significant reduction in aPDT group. Benefit in immunomodulatory response.
Skurska et al. (2015) [30]	Parallel-group RCT/36 patients/three sites with PD ≥ 6 mm, Aggressive periodontitis	SRP + PDT (18 patients)/SRP + antibiotics (18 patients)	660 nm + MB (10 mg/mL)	Three minutes incubation time, wash with saline, upward movement, 60 s irradiation per pocket/1 session: day 0	6 months	Control group significant reduction in MMP-8 No significant difference between groups in MMP-9
Carvalho et al. (2015) [31]	Parallel-group RCT/34 patients/four sites with residual pockets with PD ≥ 5 mm, Chronic periodontitis	SRP + PDT (18 patients)/SRP (16 patients)	660 nm + MB (0.1 mg/mL)	Five minutes incubation time, wash with water, 40 mW, 90 s irradiation per pocket, 90 J/cm^2^ dose, power meter used/1 session: day 45	12 months	No significant difference between groups in PD, BOP, CAL, PI
Alwaeli et al. (2015) [32]	Split-mouth RCT/16 patients/one tooth with attachment loss ≥ 4 mm in every quadrant, Chronic periodontitis	SRP + PDT/SRP	660 nm + MB (10 mg/mL)	One to three minutes incubation time, 60 mW, 6 sites per tooth, 10 s irradiation per site/1 session: day 0	12 months	PDT + SRP group significant difference in PD, CAL, BOP
Mueller et al. (2015) [33]	Split-mouth RCT/27 patients/one site in each quadrant with residual pockets with PD ≥ 4 mm, Chronic Periodontitis	SRP + PDT/SRP	670 nm + MB (0.05 mg/mL)	One minute incubation time, 280 mW, 60 s irradiation per pocket, diffusor tip/2 sessions: day 0, 7	6 months	No significant difference between groups in PD, BOP, CAL, total bacterial count
Betsy et al. (2014) [34]	Parallel-group RCT/88 patients/pockets with PD: 4–6 mm at least in two quadrants, Chronic periodontitis	SRP + PDT (44 patients)/SRP (44 patients)	655 nm + MB (10 mg/mL)	Three minutes incubation time, wash with water, 60 mW/cm^2^, 200 μm tip, 60 s irradiation per pocket/1 session: day 0	6 months	PDT + SRP group significant difference in PD, CAL No significant difference between groups in halitosis
Luchesi et al. (2013) [35]	Parallel-group RCT/37 patients/one class II furcation with PD ≥ 5 mm and BOP, Chronic periodontitis	SRP + PDT (16 patients)/SRP + MB alone (21 patients)	660 nm + MB (10 mg/mL)	One minute incubation time, wash with water, 60 mW, 600 μm tip, 60 s irradiation per pocket, upward movement, 129 J/cm^2^ dose/1 session: day 0	6 months	SRP + PDT group: significant difference in BOP, P.g, T.f and IL-1β reduction No significant difference between groups in PD, CAL, A.a., cytokines
Balata et al. (2013) [36]	Split-mouth RCT/22 patients/one pocket with PD ≥ 7 mm, one pocket with PD ≥ 5 mm and BOP on each side, Severe chronic periodontitis	SRP + PDT/SRP	660 nm + MB (0.05 mg/mL)	Two minutes incubation time, 100 mW, 9 J, 600 μm tip, 90 s irradiation per pocket, 320 J/cm^2^ dose, powermeter used, transgingival, calculated distance must be 3 mm/1 session: day 0	6 months	No significant difference between groups in PD, CAL, GI, BOP, GR
Cappuyns et al. (2012) [37]	Split-mouth RCT/32 patients/one site in each quadrant with residual pockets with PD ≥ 4 mm and BOP, Chronic periodontitis	SRP + PDT (1)/SRP + 810 nm (2), SRP (3)	660 nm + MB (0.1 mg/mL)	One minute incubation time, wash with water, 40 mW, 60 s irradiation per pocket/1 session: day 0	6 months	No significant difference between groups in PD, BOP, REC and A.a., P.g., T.f., T.d.
Filho et al. (2012) [38]	Split-mouth RCT/12 HIV patients/one site in each quadrant with PD ≥ 4 mm and BOP, Chronic periodontitis	SRP + PDT/SRP	660 nm + MB (0.1 mg/mL)	Five minutes incubation time, 30 mW, spot size 0.07 cm^2^, 133 s irradiation per point (3 buccal—3 lingual), transgingival use/1 session: day 0	6 months	SRP + PDT significant difference in PD, CAL No significant difference between groups in A.a., P.g., T.f.

**Table 2:** Studies of aPDT in periimplantitis.

Citation [ref]	Type of Study/Number of Samples/Pocket Depth	Test/Control Groups	Laser + PS Used (PS Concentration)	aPDT Protocol/Number of Sessions	Follow-Up	Outcome
Albaker et al. (2018) [39]	Parallel-group RCT/24 patients/implants with PD ≥ 5 mm and BOP, Peri-implantitis	OFD + aPDT (11 patients)/OFD (13 patients)	670 nm + MB (0.05 mg/mL)	Ten seconds incubation time, 150 mW, 600 μm tip, 60 s irradiation per pocket/1 session: day 0	12 months	No significant difference between groups in PD, BOP, MBL
Abduljabbar (2017) [40]	Parallel-group RCT/60 prediabetic patients/implants with PD ≥ 4 mm and BOP, Peri-implantitis	MD + aPDT/MD	660 nm + MB (10 mg/mL)	Two minutes incubation time, wash with H_2_O_2_ 3%, diffusor tip, 100 mW, 10 s irradiation per pocket/1 session: day 0	6 months	No significant difference between groups in PD, BOP
Romeo et al. (2016) [41]	Parallel-group RCT/40 patients/at least one implant site with PD ≥ 4 mm and BOP and suppuration, Peri-implantitis	MD + aPDT (63 implants)/MD (59 implants)	670 nm + MB (10 mg/mL)	One minute incubation, wash with water, 75 mW/cm^2^, 5 J, 600 μm tip, diffusor tip, 60 s irradiation per pocket, total 1592 J/cm^2^, 25.54 W/cm^2^/1 session: day 0	6 months	MD + aPDT showed better results in PD, BOP No *p*-value available
Bassetti et al. (2014) [42]	Parallel-group RCT/40 patients/at least one implant with PD: 4–6 mm and bone loss: 0.5–2 mm, Initial peri-implantitis	MD + aPDT/MD + local minocycline	660 nm + MB (10 mg/mL)	Three minutes incubation time, wash with H_2_O_2_ 3%, 100 mW, diffusor tip, 10 s irradiation per pocket/2 sessions: day 0, 7	12 months	No significant difference between groups in PD, CAL, REC, BOP

**Table 3:** Studies of aPDT in endodontics.

Citation [ref]	Type of Study/Number of Samples	Test/Control Groups	Laser + PS Used (PS Concentration)	aPDT Protocol/Number of Sessions	Follow-Up	Outcome
Coelho et al. (2019) [43]	Parallel-group RCT/60 patients/single-rooted teeth with fully developed apices, no probing and no mobility Rubber dam used	aPDT + RC tx (30 patients)/RC tx (30 patients) Both groups received MB for 2 min	660 nm + MB (0.5 mg/mL)	Two minutes incubation time, 100 mW, 180 s irradiation in vertical motion, 18 J, 600 J/cm^2^/1 session: day 0	7 days	aPDT + RC tx group showed significant difference in VAS score (lower) after 24 h and 72 h After 7 days no pain and no flare-up in both groups
de Miranda et al. (2018) [44]	Parallel-group RCT/16 patients/mandibular molars with apical periodontitis Rubber dam used	aPDT+RC tx (16 molars)/RC tx (16 molars) Both groups received Ca(OH)_2_ for 7 days before obturation	660 nm + MB (25 mg/mL)	Five minutes incubation time, 100 mW, 300 s irradiation in vertical motion, 300 μm tip/1 session: day 0	6 months	Clinically no significant difference, (symptoms and bacteria counts) Radiographically significant better healing
Garcez et al. (2015) [45]	Repeated measures/28 teeth with periapical periodontitis and apical bone lesion Microbiological samples: 1. after access of bone lesion 2. after conventional surgery 3. after aPDT	Conventional apical surgery + aPDT Sampling before + after aPDT	660 nm + MB (19 mg/mL)	Three minutes incubation time, 40 mW, 180 s irradiation time, 7.2 J, 200 μm tip/1 session: day 0 Additionally aPDT in the surgical cavity	Bacteria before/after Radiographs 3 years	Bacteria reduction: Conventional therapy 3.5 log surgery + aPDT 5 log (significant) Radiographic area reduction 78% (surgery + aPDT)
Juric et al. (2014) [46]	Repeated measures/21 teeth with periapical periodontitis, endodontic retreatment (endo ≥ 2 years), apical bone lesion 3 × 3 mm Microbiological samples: 1. after access of canal 2. after endo re-treatment 3. after aPDT Rubber dam used	Conventional endo re-treatment + aPDT Sampling before + after aPDT	660 + MB (10 mg/mL)	Two minutes incubation time, wash with distilled water, dry, 100 mW, 60 s irradiation time, 450 μm diffusor tip/1 session: day 0	Bacteria before/after	Chemomechanical preparation + aPDT vs. chemome-chanical preparation alone, significant difference in bacteria: Gram-positive (*p* = 0.02) Gram-negative (*p* = 0.005) facultative anaerobes (*p* = 0.013) obligate anaerobes (*p* = 0.007)
Garcez et al. (2010) [47]	Repeated measures/30 teeth of 21 patients with periapical periodontitis, endo retreatment previously with antibiotic resistance and apical bone lesion. Microbiological samples: 1. after access of canal 2. after endo re-treatment 3. after aPDT Rubber dam used	Conventional endo re-treatment + aPDT Sampling before + after aPDT Placing Ca(OH)_2_ for 7 days and then second aPDT session without sampling	660 nm + polyethylenimine chlorin(e6) (3.6 mg/mL)	Two minutes incubation time, wash with distilled water, dry 40 mW, 240 s irradiation time, 9.6 J, 200 μm tip, spiral movement/1 session: day 0	Bacteria before/after	The combination of endodontic therapy and aPDT killed all 9 multi-drug resistant bacterial species found in root canal infections No *p*-values available

**Table 4:** Studies aPDT in caries disinfection.

Citation [ref]	Type of Study/Number of Samples	Test/Control Groups	Laser + PS Used (PS Concentration)	aPDT Protocol/Number of Sessions	Follow-Up	Outcome
Alves et al. (2019) [48]	Split mouth RCT/20 patients (6–8 yrs)/occlusal surfaces homologous primary molars (20 teeth per group) (microbiological repeated measurements before/after) Rubber Dam used	Selective caries removal + aPDT/Selective caries removal Deep restoration Dycal and Ketac Molar in both groups	660 nm + MB (0.05 mg/mL)	Five minutes incubation time, wash with water, 100 mW, 180 s irradiation time, 640 J/cm^2^/1 session: day 0	6 months	After caries removal S.mutans 76% reduction (*p* = 0.04) After caries removal + aPDT S.mutans 92.6% reduction (*p* = 0.01) *p* < 0.05 between groups, no secondary caries in either group
Bargrizan et al. (2019) [49]	Parallel control RCT/56 patients (5–6 y) severe early childhood caries (Salivary S.mutans)	aPDT (14 patients)/TBO alone (14 patients), Laser alone (14 patients), Negative control (14 patients)	633 nm + TBO (0.1 mg/mL)	Kept in mouth for 5 min incubation time, spit, 20 mW, 5 min total irradiation (60 s tongue 60 s palate 90 s maxilla buccal mucosa 90 s mandibula buccal mucosa, klo4 output nozzle 1 cm^2^ area, 6 J/cm^2^/2 sessions: day 0, 3	2 weeks	Significant reduction in Salivary S.mutans in test group compared to all groups. Before second intervention S.mutans levels rising. Two interventions advisable
Ornellas et al. (2018) [50]	Microbiological repeated measurements/18 primary molars	Selective caries removal + aPDT/Selective caries removal Sampling before + after aPDT	660 nm + MB (0.1 mg/mL)	Five minutes incubation time, removal with sterile cotton, 100 mW, 90 s irradiation time, 3 mm^2^ spot, 300 J/cm^2^/1 session: day 0	Bacteria before/after	Reduction of log1 in Strep spp., Lactobacillus spp. and mutans streptococci Not significant
Steiner-Oliveira et al. (2015) [51]	Parallel-control RCT/32 patients (5–7 y) with partial caries removal in primary molars Rubber Dam used	aPDT (10 patients)/LED aPDT (10 patients)/CHX (12 patients) Sampling before/after partial caries removal	660 nm + MB (0.1 mg/mL)	Five minutes incubation time, wash with water, 100 mW, 90 s irradiation time, 320 J/cm^2^, Powermeter used/1 session: day 0	12 months	No significant difference between groups aPDT group: Log1 reduction in total bacteria count
Guglielmi et al. (2011) [52]	Microbiological repeated measurements/26 permanent molars Rubber Dam used	Selective caries removal + aPDT/Selective caries removal Sampling before + after aPDT	660 nm + MB (0.1 mg/mL)	Five minutes incubation time, no wash, 100 mW, 0.028 cm^2^ spot size, 9 J, 90 s irradiation, perpendicular to occlusal surface, one point to the center, 320 J/cm^2^, Power meter used/1 session: day 0	Bacteria before/after	Log_10_ reduction: 1.38 for mutans streptococci (*p* < 0.0001), 0.93 for Lactobacillus spp. (*p* < 0.0001), 0.91 for total viable bacteria (*p* < 0.0001)

**Table 5:** Studies with aPDT on Candida and halitosis.

Citation [ref]	Type of Study/Number of Samples	Test/Control Groups	Laser + PS Used (PS Concentration)	aPDT Protocol/Number of Sessions	Follow-Up	Outcome
Afroozi et al. (2019) [53]	Parallel-control RCT/56 patients with denture stomatitis (candida spp)	aPDT + Nystatin (28 patients)/Nystatin (28 patients) Both groups received nystatin tx 3 times per day for 15 days	810 nm + ICG (1 mg/mL)	Palatal application 10 min incubation time, no wash, 30 s irradiation time per point, 56 J/cm^2^/2 sessions: day 0, 7 (tx of denture not mentioned)	60 days	aPDT + nystatin group significant difference in candida CFU reduction After 15 days *p* = 0.013 After 60 days (*p* < 0.0001) Significant difference in reduction in lesion extension after 15 days *p* = 0.005 and in Newton’s classification (*p* = 0.007) after 60 days
de Senna et al. (2018) [54]	Parallel-control RCT/36 patients with denture stomatitis (candida spp)	aPDT (18 patients)/Miconazol (18 patients)	660 nm + MB (0.45 mg/mL)	Palatal + prosthesis: 10 min incubation time, no wash, 100 mW, 280 s irradiation time per cm^2^, dose 28 J/cm^2^/8 sessions: twice a week for 4 weeks	30 days	aPDT group significant reduction in erythema after 15 days (after 30 days no significant difference) No difference in candida CFU reduction
da Mota et al. (2016) [55]	Parallel-control RCT/46 patients with halitosis	aPDT (15 patients)/aPDT + tongue scraper (15 patients), tongue scraper alone (16 patients)	660 nm + MB (0.05 mg/mL)	Five minutes incubation time, no wash, 100 mW, 90 s irradiation time per point (6 points), 1 cm distance from each other, 9 J, fluence 320 J/cm^2^, irradiance 3.5 W/cm^2^, spot area 0.028 cm^2^, power meter used/1 session: day 0	7 days	aPDT significantly better immediate CFU results No significant differences in CFU or H_2_S results between groups after 7 days

**Table 6:** Studies with aPDT in Oral Lichen Planus.

Citation [ref]	Type of Study/Number of Samples	Test/Control Groups	Laser + PS Used (PS Concentration)	aPDT Protocol/Number of Sessions	Follow-Up	Outcome
Mirza et al. (2018) [56]	Parallel-control RCT/45 patients with erosive atrophic OLP tongue, buccal mucosa ≤3 cm	aPDT (15 patients)/LLLT (15 patients), Topical corticosteroid: dexamethasone + nystatin (15 patients)	630 nm + TBO (1 mg/mL)	Ten minutes incubation time, no wash, 10 mW, 10 mW/cm^2^, 150 s irradiation time per point, spot size 1 cm^2^, fluence 1.5 J/cm^2^/8 sessions: 2 times weekly for a month	7 days after completion of tx	Efficacy index: aPDT significant different compared to LLLT (*p* = 0.001) and corticosteroid group (*p* = 0.001) Pain control (VAS): Control group significantly better. Corticosteroids still gold standard in tx of clinical signs and symptoms
Mostafa et al. (2017) [57]	Parallel-control RCT/20 patients with oral erosive lesions	aPDT (10 patients)/Topical corticosteroid: triamcinolone (10 patients)	660 nm + MB (50 mg/mL)	Five minutes incubation time (gargle), no wash, 100–130 mW/cm^2^, 70 s irradiation time/8 sessions: Once a week for two months	2 months after completion of tx	aPDT group: VAS and lesion size decreased significantly in all follow up sessions until 2 months
Jajarm et al. (2015) [58]	Parallel-control RCT/25 patients with erosive atrophic OLP tongue, buccal mucosa ≤3 cm	aPDT (11 patients)/Topical corticosteroid: dexamethasone + nystatin (14 patients)	630 nm + TBO (1 mg/mL)	Ten minutes incubation time, no wash, 10 mW, 10 mW/cm^2^, 150 s irradiation time per point, spot size 1 cm^2^, dose 1.5 J/cm^2^/8 sessions: 2 times weekly for a month	4 weeks after completion of tx	Pain control (VAS) and Efficacy Index: Control group significantly better. No relapse (100% control group 72.7% aPDT group)

**Table 7:** Study of aPDT in healing pericoronitis.

Citation [ref]	Type of Study/Number of Samples	Test/Control Groups	Laser + PS Used (PS Concentration)	aPDT Protocol/Number of Sessions	Follow-Up	Outcome
Eroglu et al. (2019) [59]	Parallel-control RCT/40 patients with pericoronitis region of mandibular third molars	aPDT + Amoxicillin (20 patients)/Amoxicillin (20 patients) 2 Biopsies: day 0 and day of extraction-day 2	810 nm + ICG (0.1 mg/mL)	Incubation time unknown, no wash, 300 mW, 40 s irradiation time per area (operculum, distal, buccal and lingual pockets, 200 μm tip/2 sessions: day 0, 1	7 days	aPDT group: Histologically significantly better for inflammatory cell scores Day 6 (4 days after surgery): aPDT VAS = 0 vs control VAS = 1 statistically significant (but not clinical)

**Table 8:** Risk of bias assessment results.

Citation [ref]	Randomization	Sample Size Calculation and Required Number Included	Baseline Situation Similar	Blinding	Parameters of Laser Use Described Appropriately and Calculations Correct	Power Meter Used	Numerical Results Available (Statistics)	No Missing Outcome Data	All Samples/Patients Completed the Follow-Up	Correct Interpretation of Data	Total Score/10
**Periodontitis**											
Grzech-Leśniak et al. (2019) [22]	yes	no	yes	no	yes	no	yes	yes	yes	yes	7
Gandhi et al. (2019) [23]	yes	yes	yes	yes	no	no	yes	yes	yes	yes	8
Hill et al. (2019) [24]	yes	yes	yes	yes	yes	no	yes	yes	yes	yes	9
Bechara et al. (2018) [25]	yes	yes	yes	yes	no	no	yes	yes	yes	yes	8
Theodoro et al. (2018) [26]	yes	yes	yes	yes	yes	no	yes	yes	yes	yes	9
Segarra et al. (2017) [27]	yes	yes	no	yes	no	no	yes	yes	yes	yes	7
Tabenski et al. (2017) [28]	yes	yes	yes	yes	yes	no	yes	yes	yes	yes	9
Da Cruz Andrade et al. (2017) [29]	yes	no	yes	yes	yes	yes	yes	yes	yes	yes	9
Skurska et al. (2015) [30]	yes	no	no	yes	no	no	yes	yes	yes	yes	6
Carvalho et al. (2015) [31]	yes	yes	yes	yes	yes	yes	yes	yes	yes	yes	10
Alwaeli et al. (2015) [32]	yes	no	yes	yes	no	no	yes	yes	yes	yes	7
Mueller et al. (2015) [33]	yes	no	yes	yes	no	no	yes	yes	yes	yes	7
Betsy et al. (2014) [34]	yes	yes	yes	yes	no	no	yes	yes	yes	yes	8
Luchesi et al. (2013) [35]	yes	yes	yes	yes	no	no	yes	yes	yes	yes	8
Balata et al. (2013) [36]	yes	yes	yes	yes	yes	yes	yes	yes	yes	yes	10
Cappuyns et al. (2012) [37]	yes	yes	yes	yes	no	no	yes	yes	yes	yes	8
Filho et al. (2012) [38]	yes	yes	yes	yes	yes	no	yes	yes	yes	yes	9
**Peri-Implantitis**											
Albaker et al. (2018) [39]	yes	no	no	yes	yes	no	yes	yes	yes	yes	7
Abduljabbar (2017) [40]	yes	no	yes	no	no	no	yes	yes	yes	yes	6
Romeo et al. (2016) [41]	yes	no	yes	no	yes	no	no	yes	yes	yes	6
Bassetti et al. (2014) [42]	yes	no	yes	yes	no	no	yes	yes	yes	yes	7
**Endo**											
Coelho et al. (2019) [43]	yes	yes	yes	yes	yes	no	yes	yes	yes	yes	9
de Miranda et al. (2018) [44]	yes	yes	yes	yes	yes	no	yes	yes	yes	yes	9
Garcez et al. (2015) [45]	yes	no	yes	no	no	no	yes	yes	yes	yes	6
Juric et al. (2014) [46]	yes	no	yes	yes	yes	no	yes	yes	yes	yes	8
Garcez et al. (2010) [47]	yes	no	yes	yes	yes	no	no	yes	yes	yes	7
**Caries**											
Alves et al. (2019) [48]	yes	yes	yes	no	yes	no	yes	yes	yes	yes	8
Bargrizan et al. (2019) [49]	yes	yes	yes	yes	yes	no	yes	yes	yes	yes	9
Ornellas et al. (2018) [50]	yes	no	yes	yes	yes	no	yes	yes	yes	yes	8
Steiner-Oliveira et al. (2015) [51]	yes	no	yes	yes	yes	yes	yes	yes	yes	yes	9
Guglielmi et al. (2011) [52]	yes	yes	yes	no	yes	yes	yes	yes	yes	yes	9
**Candida/Halitosis**											
Afroozi et al. (2019) [53]	yes	no	yes	yes	no	no	yes	yes	yes	yes	7
de Senna et al. (2018) [54]	yes	no	yes	no	no	no	yes	yes	yes	yes	6
da Mota et al. (2016) [55]	yes	no	yes	yes	yes	yes	yes	yes	yes	yes	9
**OLP**											
Mirza et al. (2018) [56]	yes	no	yes	yes	yes	no	yes	no	no	yes	6
Mostafa et al. (2017) [57]	yes	no	yes	no	no	no	yes	yes	yes	yes	6
Jajarm et al. (2015) [58]	yes	no	yes	no	yes	no	yes	yes	yes	yes	7
**Healing**											
Eroglu et al. (2018) [59]	yes	no	yes	yes	no	no	yes	yes	yes	yes	7

**Table 9:** Studies with methylene blue (MB). * Values predominantly applied.

	MB-Perio/Peri-Implantitis 8 Papers	MB-Endo 3 Papers	MB-Caries 4 Papers	MB-Halitosis 1 Paper
Photosensitizer concentration (mg/mL)	0.05–10 10 *	5, 10, 25	0.05–0.1 0.1 *	0.05
Incubation time (min)	1–5	2, 5, 2 *	5	5
Power (mW)	60–150 100 *	100	100	100
Irradiation time (s)	48–133 60 * or 90 *	60, 180, 300	90–180 90 *	90
Tip (μm)	200–600 600 *	200, 300, 450	1900	1900
Number of sessions	1–3 1 *	1	1	1

**Table 10:** Studies with toluidine blue (TBO).

	TBO-Perio 1 Paper	TBO-Caries 1 Paper	TBO-Olp 2 Papers
Photosensitizer concentration (mg/mL)	1	0.1	1
Incubation time (min)	1	5	10
Power (mW)	200	20	10
Irradiation time (s)	30	90	150
Tip/spot size	800 μm diffusor	1 cm^2^	1 cm^2^
Number of sessions	3	2	8

**Table 11:** Single study with indocyanine green (ICG).

	ICG-Perio 1 Paper
Photosensitizer concentration (mg/mL)	0.1
Incubation time (min)	1
Power (mW)	100
Irradiation time (s)	20
Tip (μm)	300
Number of sessions	1

**Table 12:** Single study with polyethyleneimine and chlorin(e6) conjugate (PEI-ce6).

	PEI-ce6-Endo 1 Paper
Photosensitizer concentration (mg/mL)	3.6
Incubation time (min)	2
Power (mW)	40
Irradiation time (s)	240
Tip (μm)	200
Number of sessions	1

**Table 13:** Ideal reporting required for aPDT treatment regimen parameters.

Photosensitizer	Laser		
Type	Power	Tip Diameter	Trans-gingival Use or Not
Concentration	Emission Mode	Diffusor Tip or Not	Energy Distribution
Incubation Time	Irradiation Time	Tip-To-Tissue Distance	Speed of Movement
Wash/No Wash before Illumination	Total Energy Delivered	Spot Size at Tissue	
